# Frequency and types of alternative breeding strategies employed by nesting American black ducks in North Carolina

**DOI:** 10.1371/journal.pone.0278905

**Published:** 2023-02-21

**Authors:** Philip Lavretsky, Amanda Hoyt, Vergie M. Musni, Doug Howell, Christopher K. Williams

**Affiliations:** 1 Department of Biological Sciences, University of Texas at El Paso, El Paso, Texas, United States of America; 2 Department of Entomology and Wildlife Ecology, University of Delaware, Newark, Delaware, United States of America; 3 North Carolina Wildlife Resources Commission, Edenton, North Carolina, United States of America; National Cheng Kung University, TAIWAN

## Abstract

Although most birds are considered to be at least partially monogamous, molecular evidence continues to uncover that many species can have multiple sexual mates. Many species of Waterfowl (Order Anseriformes) consistently deploy alternative breeding strategies, and although cavity nesting species have been well studied, few attempts to understand rates of alternative breeding strategies exist in the Anatini tribe. Here, we assay mitochondrial DNA and thousands of nuclear markers across 20 broods of American black ducks (*Anas rubripes*; “black duck”) that included 19 females and 172 offspring to study population structure as well as types and rates of secondary breeding strategies in coastal North Carolina. First, we report high levels of relatedness among nesting black ducks and offspring and while 17 (of 19) females were of pure black duck descent, three were found to be black duck x mallard (*A*. *platyrhynchos*) hybrids. Next, we evaluated for mismatched mitochondrial DNA and paternity identities across each female’s clutch to determine types and frequency of alternative or secondary breeding strategies. Although we report that nest parasitism occurred in two nests, 37% (7 of 19) of the sampled nests were multi-paternal as a result of extra-pair copulation. In addition to being part of a mix of strategies used to increase fecundity by successfully breeding females, we posit nest densities providing easier alternative mate access for males also explains high rates of extra-pair copulation among our sampled black ducks. Ultimately, however, while some proportion of females of many species engage in forms of secondary breeding strategies, we conclude that the decision to do so appears to be seasonally flexible for each individual.

## Introduction

While a large proportion of the animal kingdom was once thought to be monogamous, advancements in molecular methods have clarified that many individuals were only socially so [reviewed in Klug 1]. Advantageous of successful mate pairs to maintain monogamy stems from the relative certainty of partner access and potential reproductive success, especially in cases where dual-parental care increases the chance survival of altricial offspring [1]. However, even in 90% of bird species that were once considered minimally seasonally monogamous [2], paternity tests have confirmed that many have multiple sexual mates [3]. Alternative or secondary breeding strategies include extra–pair paternity and intraspecific brood parasitism [4, 5]. Either of these strategies can vary within a population and have been linked to ecological and genetic differences at the individual level [6]. While Bateman’s principle [7] may explain why males seek out additional mates to directly increase their fitness [i.e., sneaky males; 8], females exhibiting alternative breeding strategies do so to increase their chances of having higher quality offspring and to increase fecundity without the need of more parental investment [9–13]. Commonly employed secondary breeding strategies include extra-pair copulation [EPC; 14], nest parasitism [15], and brood amalgamation [16]. Together, the probability a pair that is socially monogamous is also sexually monogamous appears to be an individual’s choice, depending on a male’s or female’s body condition, social standing, and probability of surviving and reproducing in following years [6]. This large heterogeneity within populations makes predicting extant of populations exhibiting alternative breeding strategies nearly impossible [17].

Of avian lineages, many species of Waterfowl (Order Anseriformes) consistently deploy alternative breeding strategies [18–20]. In particular, cavity nesting species have been well documented to annually engage in forms of parasitism [i.e., nest parasitism; 13, 19, 20–22], with less evidence existing for ground nesting species such as the American black ducks (*Anas rubripes*; “black duck”). Few studies have attempted to understand rates of nest parasitism in the Anatini tribe [16, 20, 22–26], and many of them are largely anecdotal [e.g., black ducks; 27]. In general, studies conclude that the probability of deploying alternative breeding strategies among ground nesting duck species is directly related to nesting density [16, 20, 22–25]. However, there have been no attempts to directly validate and understand which secondary breeding strategies are being employed by both sexes, and at what rates among any Anatini species.

Here, we assayed mitochondrial DNA (mtDNA) and thousands of nuclear markers to study population structure and types and rates of secondary breeding strategies being employed by black ducks in coastal North Carolina. First, we established relatedness and population structure of our sample set. Moreover, hybridization between black ducks and wild mallards (*Anas platyrhynchos*) is well documented at the landscape level [28], with additional evidence of congeners interbreeding with locally released game-farm mallards in North [29] and South [30] Carolina. By including reference American black ducks, as well as wild and game-farm mallards, we are able to determine local ancestry as it compares to the larger black duck population, along with rates of hybridization. Note that game-farm mallards are domestic mallards being raised and released on shooting preserves for hunting purposes, and this practice has led to significant rates of gene flow with wild populations of ducks in North America [28–31]. In addition to increased rates of hybridization, breeding black ducks in North Carolina appear to be largely residential and have local ancestry suggesting they have been breeding in the area for some time [29]. Thus, we expected to find evidence of high relatedness along with a proportion of females and/or nests comprised of black duck x mallard (wild or game-farm) hybrids. Next, following two visual incidences of nest parasitism among breeding black ducks in the study area, females and their nests were sampled post-hatch to genetically-examine for evidence of alternative breeding strategies potentially being deployed by these ducks. Specifically, we assess the total number of mitochondrial haplotypes, as well as nuclear-based ancestry and sibship assignments among breeding females and their nests. If a nest is parasitized by an unrelated female, we expect these egg(s) to carry unique mtDNA haplotype(s) and non-sib relationships as compared to the incubating female and the rest of her eggs. For females engaging in, or forced upon, EPC, we expect multi-paternal broods to possess lowered relatedness and be assigned as half-siblings. Further, because of the high nesting density occurring on nesting islands and evidence for high year-to-year nest fidelity [29], we assessed whether a high degree of relatedness in crowded conditions could explain potential nest parasitism or other recovered forms of alternative mating strategies.

## Materials and methods

### Sampling methods

**Fig 1:**
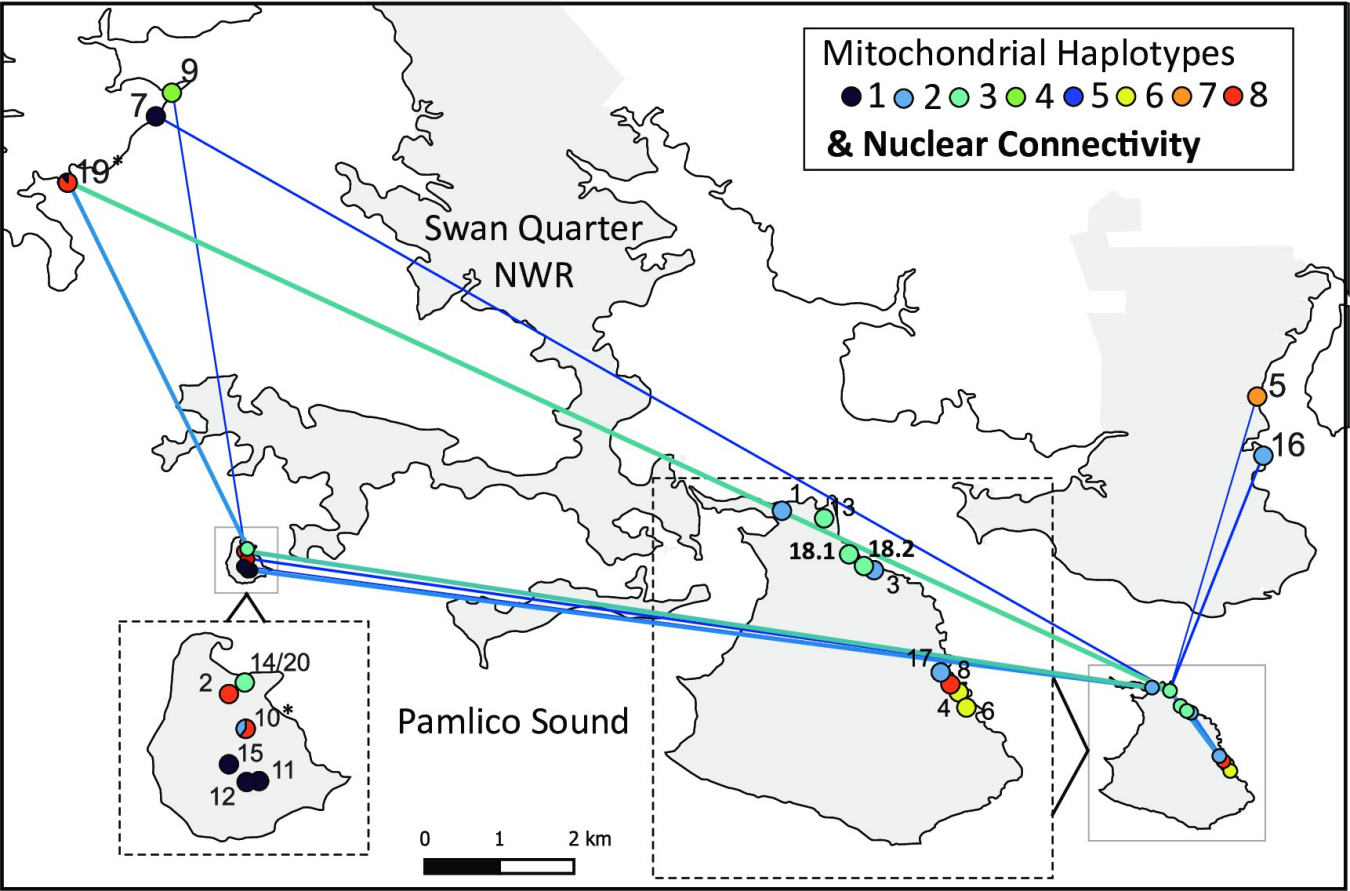
Sampling location and ID for each American black duck nest (see sample specifics in S1 Table) represented across the study area and created in ArcMap 10.7.1(Esri). An expanded view of both sampled islands and their respective nests are provided. Note nests are color coded and proportional to the mitochondrial haplotype(s) recovered in each nest; only nests 10 and 19 had >1 haplotype present and are denoted with an asterisks. Furthermore, nuclear connectivity reconstructed using 259 independent autosomal ddRAD-seq SNPs in the EDENetworks program version 2.18 [32] are overlaid across nests. The weight and color denote levels of nuclear relatedness, finding that all sampled nests are related (also see S3 Fig). The Swan Quarter National Wildlife Refuge (NWR) is shaded in grey, and the Pamlico Sound is also denoted.

We collected samples from coastal marshes in Hyde County, North Carolina (35.372° N 76.358° W) from March through July in 2020 and 2021 (Fig 1). Field work was conducted in quality black duck nesting habitat identified previously by Lawson et al. [33], and included mainland brackish marshes and natural islands with thick grassy vegetation within the Pamlico Sound. Sampled islands ranged in size from 26–271 ha and sat 0.4–1.2 km from the nearest mainland. Nest success on the mainland and natural islands were 11.11% (N = 54) and 60.53% (N = 51), respectively, with differences being due in part to greater accessibility of mainland areas to predators such as raccoons [33]. Previous research in Pamlico Sound estimated nesting density of regularly flooded marsh to be 1 nest/22 ha [33], which was corroborated by our findings on natural islands. We located active nests (i.e., from egg laying through hatching) using modified nest dragging [33, 34]. Once located, all active nests were monitored to determine stage of egg development using a combination of candling and floating [35, 36]. Once pipping was confirmed in at least one egg, all eggs were placed in a plastic mesh bag to contain the hatched ducklings, and a modified nest trap [37] was placed over the nest. On the day of hatch (i.e., within 24 hours), females and broods were captured by slowly and quietly approaching the nest and blocking the exit from the trap, preventing the female from escaping. During this time, 172 samples of offspring were obtained that included chorioallantoic egg membranes (N = 135) or web punches of chicks (N = 37). Web punches were collected using a standard size single hole-punch on the outer edge of the foot [38]. Only half of the hole punch was filled resulting in samples being ~14 mm^2^. A total of 19 nesting females were captured and blood was collected from the tarsus [39]. Females were also marked with a federal leg band, weighed (g) and aged based on primary and tertial coverts [40]. Only after a female blood sample was secured was the associated brood sampled. In one instance two broods from the same female were sampled resulting in 20 physical chicks. All hatched and inviable eggs were placed into labeled Ziploc bags, while we placed web-punches and blood samples into labeled 1.5 ml microcentrifuge tubes filled with 80% ethanol or blood preservation buffer [41], respectively, and then stored at -80°C. Capture and sampling were done in accordance with federal and state laws under the North Carolina Wildlife Resources Commission’s Federal Bird Banding Permit (#06557) and University of Delaware Animal Use and Care (#1356–2021).

### DNA isolation

We extracted genomic DNA from the 191 chorioallantoic membranes, web punches, or blood using a DNeasy Blood and Tissue kit following the manufacturer’s protocol (Qiagen, Valencia, CA, USA). DNA quality was based on the presence of high molecular weight band visualized using gel electrophoresis and with a 1% agarose gel, and quantified using a Qubit 3 Flourometer (Invitrogen, Carlsbad, CA) to ensure a minimum concentration of 20 ng/μL. While all samples were Sanger sequenced for mtDNA, only samples possessing high molecular bands were attempted for double digest restriction-site associated DNA (ddRAD-seq) library preparation [42].

### Sex determination

We determined sex by amplifying homologous CHD gene found on both sex chromosomes of birds [43]. In short, the amplified CHD gene found on the Z- versus W-sex chromosomes differ by several bases resulting in the PCR amplification and gel electrophoresis of one versus two bands in the homogametic (i.e., males = ZZ) versus the heterogametic (i.e., females = ZW) sexes, respectively. Primers for PCR amplification were based on Çakmak et al. [43], but we re-optimized PCR mixture and thermocycler conditions. First, PCR reactions comprised 1.5 μL of template DNA (≥10 ng/μl), 2x GoTaq Green Master Mix (Promega), and 1.0 nM of each primer, in a total volume of 15 μL and conducted using an Eppendorf Mastercycler (ep*gradient*) thermocycler following a touch-down protocol that included an initial denaturation at 94°C for four minutes, followed by a single 94°C cycle for 30 seconds, before annealing for another 45 seconds starting at 57°C decreasing by one degree each cycle to 50°C, and a final 45 second extension at 72°C. This touch-down PCR protocol was followed by 30 cycles of 30 seconds at 94°C, 45 seconds at 50°C, and 45 seconds at 72°C, with a final extension at 72°C for five minutes. Amplification was then verified using gel electrophoresis with a 4% agarose gel.

### Mitochondrial DNA

The mtDNA control region was assessed across samples. We used primers L78 and H774 to polymerase chain reaction (PCR) amplify and sequence 625 base pairs of the mtDNA control region [44, 45] following Sanger Sequencing methods described in Lavretsky et al. [46]. PCR products were visualized via agarose electrophoresis and then purified using ExoSAP-IT (ThermoFisher). Purified PCR product was then sequenced using the L78 primer on a 3130XL Genetic Analyzer (Applied Biosystems, Waltham, MA, USA) at the University of Texas at El Paso, Border Biomedical Research Center’s Genomic Analysis Core Facility. We aligned and edited sequences using Sequencher version 4.8 (Gene Codes, Ann Arbor, MI, USA). All sequences have been submitted to GenBank (accession numbers OP945955—OP946145). Prior to analyses, overlapping mtDNA control region sequences for reference wild mallard, game-farm mallard, and black duck reference samples were included [28, 31, 46, 47]. A mtDNA haplotype network was inferred using a median-joining algorithm in the POPART v. 1.7 program [48]. Note that among mallard-like ducks, there are two divergent mtDNA haplogroups: Old World (OW; Eurasian origin) A and New World (NW; North American origin) B [47, 49, 50]. In general, the presence of OW A mtDNA among wild North American birds is due to hybridization with game-farm mallards that are of Eurasian stock [31]. Thus, in addition to using mtDNA to evaluate the maternal structure on the landscape, we determined the number of nests carrying OW A mtDNA haplotypes as a proxy for game-farm mallard gene flow rates in the area.

### ddRAD-seq library preparation and sequencing

For 114 (of 173) samples that possessed high-molecular weight bands, we followed procedures presented by Lavretsky et al. [51] to create multiplexed ddRAD-seq fragment libraries. In short, we enzymatically fragmented genomic DNA using SbfI and EcoRI restriction enzymes, and ligated Illumina TruSeq compatible barcodes that permitted future de-multiplexing. All library were pooled in equimolar concentrations, and 150 base pair (bp), single-end (SE) sequencing was completed on an Illumina HiSeq X at Novogenetics LTD (Sacramento, CA). Illumina reads were deposited in NCBI’s Sequence Read Archive (SRA; http://www.ncbi.nlm.nih.gov/sra; SRA data PRJNA907259).

We used the *ddRADparser*.*py* script of the BU ddRAD-seq pipeline (DaCosta and Sorenson 2014) to de-multiplex raw Illumina reads based on perfect barcode/index matches. As with mtDNA, previously published ddRAD-seq raw sequence data generated using the same protocols were included in alignments and subsequent analyses, serving as reference wild mallard, black ducks [28], and game-farm mallards [31]. All sequences were first trimmed or discarded for poor quality using program Trimmomatic [52], and then the remaining sequences were aligned to a chromosomal-level reference wild mallard genome [53] using the Burrows Wheeler Aligner v. 07.15 [bwa; 54]. Samples were then sorted and indexed in Samtools v. 1.7 [52] and combined using the “mpileup” function with the following parameters “-c–A -Q 30 -q 30.” All steps through “mpileup” were automated using a custom in-house Python script [Python scripts available at https://github.com/jonmohl/PopGen; see 31]. Next, we used VCFtools v.0.1.15 [55] to filter VCF files for any base-pair missing >5% of samples that also included a minimum base-pair depth of 5X (i.e., 10X per genotype) and quality per base PHRED scores of ≥30. Only autosomal loci were used in population genetics, relatedness, and sibship analyses.

### Relatedness and population genetics

Prior to analyses, we used PLINK v. 1.9 [56] to ensure that singletons (i.e., minimum allele frequency [maf] = 0.0056) and any SNP missing >5% of data across samples were excluded in each dataset. Additionally, we identified independent SNPs by conducting pair-wise linkage disequilibrium (LD) tests across ddRAD-seq autosomal SNPs (—indep-pairwise 2 1 0.5) in which 1 of 2 linked SNPs are randomly excluded if we obtained an LD correlation factor (*r*^2^) > 0.5. We conducted all analyses without *a priori* information on population or species identity.

Given that we expected high relatedness among North Carolina samples [29], we first obtained co-ancestry assignments across independent bi-allelic nuclear ddRAD-seq SNPs in the program fineRADstructure [57]. We ran fineRADstructure with a burn-in of 100,000 iterations, followed by 100,000 Markov chain Monte Carlo iterations, followed by tree building using default parameters. Results were visualized using the R scripts fineradstructureplot.r and finestructurelibrary.r (R Core Team 2020). Additionally, we calculated a pair-wise sample relatedness matrix using the relatedness (—relatedness) function as implemented in VCFtools v. 0.1.15 [55], and based on the same independent bi-allelic nuclear ddRAD-seq SNP dataset. In short, the relatedness matrix is comprised of unadjusted Ajk statistics calculated based on the methods outlined in Yang et al. [58] where pair-wise sample relatedness is scaled from 0 to 1.

Preliminary analyses of individual assignment probability estimates when including all or partial samples in the program ADMIXTURE v.1.3 [59, 60] were found to be highly confounded by the high levels of co-ancestry and relatedness within our dataset (see Results for detail). Consequently, assignment probabilities were obtained by running each sample independently against our reference set for the same independent bi-allelic nuclear ddRAD-seq SNPs. ADMIXTURE analyses were run based on a *K* population model of three, with a 10-fold cross validation, and with a quasi-Newton algorithm employed to accelerate convergence [61]. Each analysis used a block relaxation algorithm for point estimation and terminated once the change in the log-likelihood of the point estimations increased by <0.0001. Moreover, standard errors for each analysis were based on 10 bootstrap replicates. Final outputs for reference samples were based on averaging Q scores and respective standard errors across analyses. Any sample with a Q-score and standard errors overlapping ≥98% population assignment was considered as genetically pure, otherwise they were demarcated as hybrids [28]. Doing so allowed us to determine the genetic constitute of breeding females of the area and their potential mate(s). For example, if a genetically pure black duck female has a nest entirely comprised of eggs with 50:50 ancestry of black duck and wild mallard, then we can conclude the father had to have been a wild mallard. Importantly, we are able to determine whether the interspecific pairing leads to an entire (i.e., seasonal interspecific monogamy) or partial (i.e., extra-pair copulation) clutch.

Finally, pair-wise population relative differentiation (F_ST_) and per population nucleotide diversity was calculated across ddRAD-seq loci using the Pixy Program [62].

### Maternal and sibling relationships

Relationships among breeding females and each of their clutches was quantified in the program COLONY v. 2.0.6.5 [63]. Program COLONY implements full-pedigree likelihood methods to simultaneously infer sibship and parentage among individuals using multilocus genotype data. Analyses in COLONY were based on ddRAD-seq autosomal loci with <5% missingness and a minimum allele frequency of 0.5 across samples. To reduce the risks of type I error, we only reported parental, full-sibling, and half-sibling dyads with pairwise relatedness estimates that were greater than 0.2 [64]. Moreover, COLONY infers paternal lineages across samples, providing us the ability to assess whether females that were socially monogamous proved to be so with the number of sexual mates present in their assessed clutches. In addition to running an analysis in which offspring and mothers were compared, we also ran all mothers in a single analysis to determine the number of related females in the dataset.

Finally, we wanted to understand the connectivity among the 20 nest groups. To do so, we used the same SNP dataset analyzed in COLONY to create a genetic network based on a minimum spanning network (MSN) as implemented in the EDENetworks program v. 2.18 [32]. In short, EDENetworks uses percolation theory to construct a network of either individuals or populations as nodes, with the connecting edges weighted by their pairwise genetic distance (F_ST_). Analyses were done without any *a priori* information on population identity or sampling location.

## Results

### Relatedness and genetic clustering among samples

Although sufficient DNA quality and quantity required to construct ddRAD-seq libraries was obtained for 114 (of 191) samples, sufficient sequencing was obtained for 99 of these (19 females and 80 offspring). After combining all successfully sequenced samples and those acting as reference populations, we obtained a dataset of 80,035 base-pairs.

**Fig 2:**
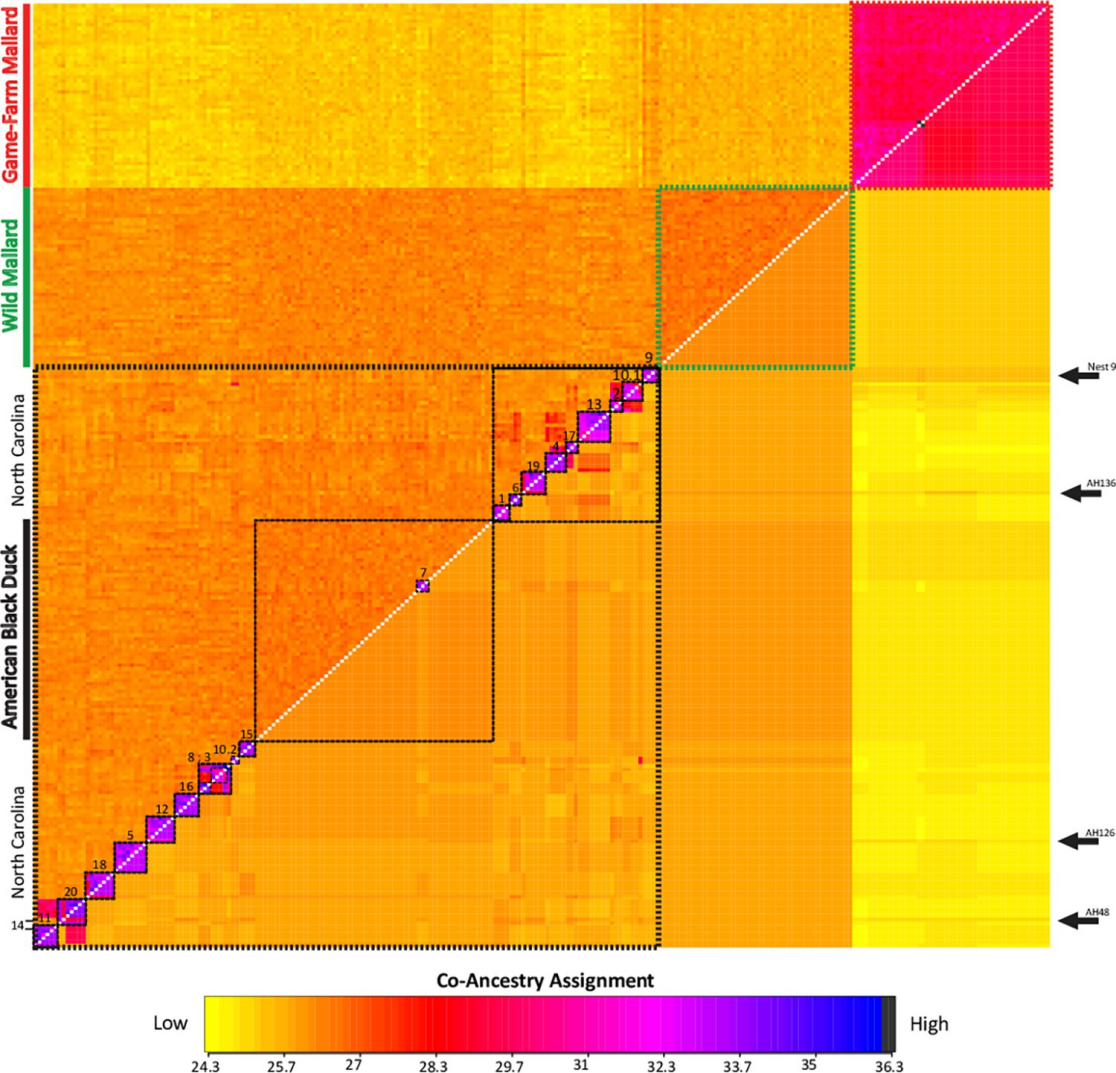
FineRADstructure individual (above diagonal) and average (below diagonal) co-ancestry coefficient matrix based on a 6,622 independent bi-allelic ddRAD-seq autosomal SNP dataset for sampled reference American black ducks, wild mallards, and game-farm mallards, and samples from North Carolina, USA, 2020–2021. Samples are color coded by reference species or population. The level of co-ancestry is color coded from low (yellow) to high (blue). The 20 genetic clusters found across the 99 North Carolina samples are numbered and correspond to their nest identity (S1 Table). Finally, arrows denote the group (i.e., nest 9) or individuals that showed higher-than average levels of co-ancestry with game-farm mallards.

First, a filtered dataset of 6,622 independent autosomal bi-allelic ddRAD-seq SNPs that met filtering criteria was used to calculate relatedness and co-ancestry across all 99 North Carolina samples. Calculating pair-wise sample Ajk statistics recovered full sibling relationships (Ajk statistic ~ 0.50) between each of the sampled females and their offspring in most cases. However, less than full sibling (Ajk statistic ranging 0.10 ~ 0.40) relationships among offspring were recovered across four of 19 nest groups (S2 Table). Moreover, substantial relatedness (Ajk statistic > 0.1) was also found between these 19 groups (S2 Table). This level of within and between group relatedness among North Carolina samples was then visualized by plotting pair-wise sample co-ancestry results from fineRADstructure with the same set of independent autosomal ddRAD-seq SNPs (Fig 2). Co-ancestry plots recovered three major genetic groups clustering samples as game-farm mallard, wild mallard, or black duck; including the reference samples as expected (Fig 2). Of the three major groups, North Carolina samples showed highest co-ancestry and clustering with reference black ducks; however, there were two evident North Carolina groups, each comprising sets of highly related individuals. In total, we identified 20 unique genetic clusters within the North Carolina samples. Importantly, each cluster was comprised of a set of related offspring and/or a single maternal sample. Thus, the co-ancestry plot not only recovered expected maternal-sibling clusters for the nests where all offspring were sampled alongside the female, but also identified a set of eggs from of un-sampled female (i.e., group 10.2). Note the high levels of co-ancestry among the different parent-offspring clusters suggesting that many of these groups are related at higher levels (i.e., cousins), and consistent with Ajk relatedness scores (S2 Table). For example, the offspring from nests 11 and 20 showed high levels of co-ancestry and Ajk relatedness scores consistent with a half-sibling relationship, suggesting these offspring had similar paternal lineages. Similarly, the high level of co-ancestry among offspring and females of nests 8 and 3 or nests 2 and 10.1 suggest the individuals of these groups are likely cousin relatedness; once again, consistent with the non-zero Ajk statistics for these comparisons as well (S2 Table). We also note that group nest 18 is in fact comprised of eggs hatched by the same female (AH10) a month apart, with the first nesting attempt on 6 April 2021 and then re-nesting on 30 May 2021; both clutches show similar co-ancestry and Ajk relatedness scores suggesting the same maternal-paternal combination in both nesting attempts.

**Fig 3:**
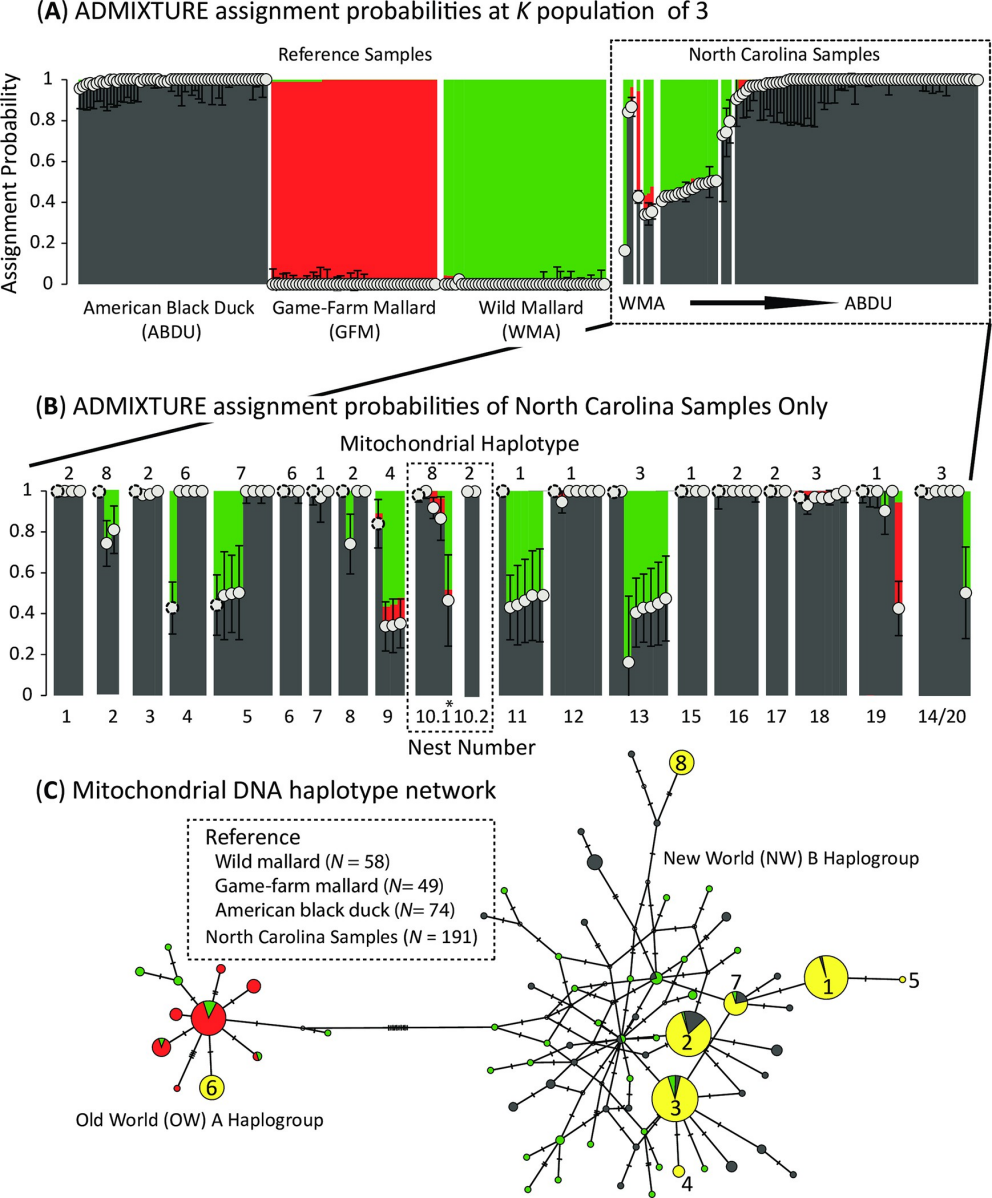
(A) Individual population assignment probability as estimated in the program ADMIXTURE for a population *K* of 3 model and based on a 16,819 independent bi-allelic ddRAD-seq autosomal SNP dataset for sampled reference American black ducks, game-farm mallards, and wild mallards, and 99 samples from North Carolina, USA, 2020–2021. We plot point ancestry and associated standard error assignment to the American black duck genetic cluster across samples. Any sample with standard error’s overlapping ≥98% assignment to the American black duck was considered as genetically pure black duck. (B) Individual assignment probabilities of North Carolina samples only re-ordered by nest identity as established in co-ancestry clustering (Fig 2). Note that any nest with an associated female is at the start of each nest group, and denoted with a dotted outline on their point estimate. The major mtDNA haplotype and nest number associated with each group is provided above and below the assignment probability plot, respectively. Finally, (C) a reconstructed haplotype network for the mitochondrial DNA (mtDNA) control region sequenced across all reference and 192 North Carolina samples. Graduated circles represent sample size and dashes along network lines represent genetic distance between and within mitochondrial haplogroups. The previously identified Old World A (OW A) and New World B (NW B) haplogroups, as well as the eight haplotypes found across North Carolina samples are denoted.

Next, compiling assignment probabilities across the sample-by-sample ADMIXTURE analyses provided estimates unbiased by the evidently high interrelatedness among North Carolina samples. For these analyses, we obtained 16,819 independent autosomal bi-allelic ddRAD-seq SNPs that met filtering criteria when combining all reference samples and one North Carolina sample at a time. Each analyses was run at a population *K* of 3, in which we recovered the expected three groups that included game-farm mallards, wild mallards, and black ducks, with all reference samples assigning to their respective genetic clusters (Fig 3A). Though most North Carolina samples were assigned to the black duck cluster (Fig 3A), ancestry generally followed nest locations (Fig 3B). Among females, 16 (of 19) were assigned as genetically pure black duck, and those of nest groups 4, 5, and 9 being outcrossed with a wild mallard. Comparing the genetic ancestry of the females and their associated offspring revealed more specific patterns. Among the 16 pure black ducks, offspring of nine nests was the result of a black duck male pairing, whereas two other nests were the pairings with wild male mallards (i.e., nests 2 & 13). The remaining five nests of black duck females appeared to be of more complex mating strategies (see COLONY results; Table 1). For the three hybrid females, their respective offspring genetic identity were consistent with two being paired with a black duck as all the offspring appear to have reverted to pure black duck ancestry (nests 4 & 5), while the other two likely mated with a wild mallard x black duck hybrid (nest 9; Table 1).

**Table 1:** Inferred maternal lineages and their associated paternal lineages that explain the genetic diversity of each brood as determined in the program COLONY are provided. Maternal species identity was inferred from their respective ADMIXTURE assignment probability (Fig 3). Paternal species identity was inferred through comparing the maternal and offspring ADMIXTURE assignment probabilities as any differences are the result of different paternal and maternal lineages. The number of sampled eggs that are explained by the pairing across broods are provided in parenthesis next to the inferred paternal lineage. Paternal lineages found in more than one pairing are denoted with asterisk. Note species’ identities included American black duck (ABDU), wild mallard (WMA), and game-farm mallard (GFM) (also see S1 Table for sample specific information).

Nest	Inferred Maternal Lineage	Inferred Maternal Species	Inferred Paternal Lineage (# of offspring)	Inferred Paternal Species
nest 7	AH1	ABDU	#9 (2)	ABDU
nest 10.1 (AH63 sired by inferred father #10)	AH2	ABDU	#10 (1)*; #11 (3)	#10 (ABDU); #11 (ABDUxWMAxGFM)
nest 2	AH3	ABDU	#12 (2)	ABDUxWMA
Inferred nest 14/20 (AH48 sired by inferred father #8)	AH4	ABDU	#7 (5); #8 (1)	#7 (ABDU); #8 (MALL)
Inferred nest 15	AH5	ABDU	#13 (3)	ABDU
Inferred nest 16	AH7	ABDU	#14 (5)	ABDU
nest 12 (AH126 has inferred father #3)	AH8	ABDU	#2 (5); #3 (1)	#2 (ABDU); #3 (ABDU)
Inferred nest 17	AH9	ABDU	#15 (2)**	ABDU
Inferred nest 18 (AH168 sired by inferred father #22)	AH10	ABDU	#1 (5); #21 (1)	#1 (ABDU); #22 (ABDU)
nest 3 (AH154 sired by inferred father #21)	AH11	ABDU	#19 (2); #20 (1)	#20 (ABDU); #21 (ABDU)
nest 4	AH13	ABxWMA	#15 (4)**	ABDU
nest 5	AH14	ABxWMA	#22 (7)	ABDU
nest 6	AH15	ABDU	#6 (2)	ABDU
nest 9	AH17	ABxWMAxGFM	#23 (3)	ABDUxWMA
nest 8	AH18	ABDU	#24 (3)	ABDU
nest 11	AH210	ABDU	#7 (5)	MALL
inferred nest 19 (AH136 sired by inferred father #16)	#1 (should be **AH16**) < 0.5 PROB	ABDU	#4 (4); #16 (1)	#4 (ABDU); #16 (GFM)
nest 1	#2 (should be **AH12**) < 0.5 PROB	ABDU	#5 (3)	#4 (ABDU)
10.2 egg dumped into nest nest 10	#3 (**unsampled female**)	ABDU	#10 (2)*	ABDU
nest 13 (AH139 inferred father #18)	#4 (should be **AH19**)	ABDU	#17 (6); #18 (1)	#17 (ABDU); #18 (ABxWM)

Finally, a total of 624 base-pairs of the mtDNA control region were successfully sequenced across all 191 North Carolina samples and when combined with reference wild and game-farm mallard sequences. Network reconstruction recovered the expected OW A and NW B mtDNA haplogroups (Fig 3C). A total of eight haplotypes were recovered among North Carolina samples, with one and seven falling into the OW A and NW B haplogroups, respectively (see S1 Table for sample specific info). Of these, the one OW A and four NW B haplogroups were not shared with any of the reference samples. Conversely, the four major NW B haplotypes recovered among North Carolina samples were shared with black ducks and/or wild mallards (Fig 3A; see S1 Table for sample specifics). Note that the one OW A haplotype found among females and offspring of nests four and six (Fig 3B) was only one mutation away from the major OW A haplotype found among reference game-farm mallards; suggesting that an un-sampled but closely related game-farm mallard maternal lineage had been introgressed in the recent past, which is consistent with previous work [29]. Offspring carrying an alternative mtDNA haplotype as compared to the laying female were found in only two nests (10 and 19; Fig 1; S1 Table). Specifically, whereas nest 19 had one (of 9), nest 10 had 4 (of 9) eggs that differed in sequence, and thus, were the result of parasitism.

### Relationships among North Carolina samples

We used 259 independent autosomal ddRAD-seq SNPs that met our missing data and minimum allele frequency criteria for COLONY and network analyses. First, only one pair of females were found to be true full sibling sisters (AH11 (nest 3) & AH18 (nest 8)), and another as half-siblings (AH2 (nest 10.1) & AH3 (nest 2); S1 Fig); both of these female combinations indeed carried the same respective mtDNA haplotype (Figs 1 & 3B; S1 Table). Next, among the offspring, COLONY identified 119 and 65 full and half sibling relationships, respectively (S2 Fig). Each of the recovered full and half sibling relationships among offspring were concordant with elevated relatedness matrix (S2 Table) and co-ancestry assignments (Fig 2). Importantly, COLONY inferred maternal and paternal lineages across offspring (S1 Table), permitting us to estimate the number of male mates per nest. A total of 20 female and 24 male lineages were inferred to explain the genetic diversity of the 80 offspring (Table 1; S1 Table). Among the 20 maternal lineages, 16 (of 19) of the known females were assigned to their respective offspring. While COLONY inferred mother-offspring relationships with < 50% probability for the remaining three sampled females, the same maternal lineage were correctly inferred to their respective clutches by COLONY (Table 1). We note that the low probability assignment of these females to their respective eggs is likely data limitation [65] as each of them clustered and showed high co-ancestry (Fig 2) and relatedness Ajk statistics (S2 Table) with their respective offspring when using the full SNP dataset. Finally, the two unique eggs found in nest 10 that did not cluster in co-ancestry plots (Fig 2), nor had the same mtDNA haplotype (Fig 3B) were inferred to be the result of a nest parasitism event by an un-sampled female and inferred paternal lineage 10 (Table 1). In fact, the inferred paternal lineage 10 was also determined siring the offspring that was a half-sibling (i.e., AH63) to the rest of the clutch; suggesting that the same male fathering the parasitized eggs also had the opportunity for extra-pair copulation with the mother of that nest.

Based on the maternal-paternal ancestry of sampled eggs per nest, we estimate that 65% of females (13 of the 20) were socially and sexually monogamous. Notably, extra-pair copulation appears to have resulted in only one egg in each of the respective nests. Thus, the mate that was socially monogamous still obtained the majority benefit towards their fecundity. Among males, we found a single inferred paternal lineage that sired the majority of eggs found in nests 11 (nest completion date 5/24) and 14/20 (nest completion date 5/16) (Table 1). This is consistent with co-ancestry assignments (Fig 2), and Ajk statistics (S2 Table) showing high relatedness among the offspring of those two nests but not the females. Given that both of these nests were completed within a week of each other and on the same Island (Fig 1), it seems that this male black duck was promiscuous while the females in these relationships were monogamous. Finally, COLONY inferred the same paternal lineage #1 for both clutches made by female AH10 that first nested on 4/6/2021 and then re-nested on 5/30/2021; once again, consistent with co-ancestry and relatedness estimates placing all offspring from both clutches as full siblings (Table 1; Fig 1; S1 Table).

Finally, the EDENetworks network analysis of the 19 known and inferred nests (i.e., excluding 10.2; Table 1) recovered a ‘star-like’ pattern of connectivity, with nest 13 being the center of it (S3 Fig). Mapping the network across geographical space further demonstrates how all the sampled nests are genetically connected, and consistent with the high relatedness found across other analyses (Fig 1).

### Sex ratios

Sex IDs were successfully obtained across offspring extractions and confirmed across female samples. Correcting for any parasitized eggs found in nests 10 and 19, the sex ratios were statistically similar to 50:50 (two-tailed t-test p-value = 0.16; S4 Fig). Moreover, excluding the unknown female that parasitized nest 10 (i.e., 10.2), the average clutch size of those nests with complete offspring count was 8.3 (range = 5–13 eggs/nest), which is consistent with general clutch sizes of black ducks [66].

### Hybridization

Putative hybrids were recovered across North Carolina samples in both fineRADstructure (Fig 2) and ADMIXTURE (Fig 3A) analyses. First, co-ancestry analyses found higher-than average assignment to the game-farm mallard cluster among individuals comprising nest 9, as well as offspring samples AH136, AH126, and AH48. No sample showed higher-than average assignment to the wild mallard group. Indeed the female and offspring of nest 9, as well as AH136, AH126, and AH48 had slight to moderate ancestry assignment to the game-farm mallard genetic cluster. However, ADMIXTURE also recovered two more females and several other offspring with substantial assignment to wild mallard (Fig 3B). Given that none of the sampled females were found to be wild or game-farm mallards, molecular contributions from wild or game-farm mallards must be via previous (e.g., nest 9) or ongoing mate-pairings. Specifically, while we do not find that a male game-farm mallard was the primary mate, we find that at least one male game-farm mallard did obtain one egg through extra-pair copulation in nest 14/20 (Table 1). Similarly, we find that two and four of the inferred males must have been wild or wild x black duck hybrids, respectively, given the composition of the mother and offspring (Table 1). Of those non-black duck males, mallards and hybrids were the primary mate in one and three of their respective events (Table 1). In the end, 15 of the 17 females inferred as pure black duck were socially monogamous with their primary mate that was also a black duck. Conversely, among the hybrid females, one and two paired with another hybrid or black duck male.

### Relative differentiation and genetic diversity

Despite the strong relatedness among the North Carolina samples, we did not find any indication the population is suffering from a lack of genetic diversity, with the range and mean of their calculated nucleotide diversity across ddRAD-seq loci being identical to reference wild mallards and black ducks (S5A Fig). Similarly, North Carolina samples, as well as the reference wild mallards and black ducks were all genetically very similar (Φ_ST_ < 0.02; S5B Fig), and as expected due to the two species’ recent ancestral history [31, 67]. Once again, the generally low relative genetic differentiation between North Carolina samples and reference black ducks and wild mallards suggests that the population had not gone through severe genetic drift.

## Discussion

### Resident American black ducks of coastal North Carolina

Here, assessing the genetic composition of American black duck females and their offspring nesting on several close natural islands within the Pamlico Sound of North Carolina (Fig 1), we find that all the individuals are to some degree genetically related, with no evidence of outside immigration contributing to the locations maternal breeding pool. In addition to high rates of co-ancestry (Fig 2) and relatedness (S2 Table), EDENetworks network analysis of the 19 known and inferred nests (i.e., excluding 10.2; Table 1) recovered a ‘star-like’ pattern of connectivity, demonstrating that all of the sampled nests are genetically connected, with none of them being of novel genetic source (Fig 1; S3 Fig). Similar to the nuclear-based network, a mtDNA network also showed low diversity and ‘star-like’ pattern as compared to the reference wild mallard and black ducks that is consistent with severe bottlenecking due to founder events by a few maternal lineages (Fig 3C). Together, the molecular data provides strong evidence of a locally breeding population of black ducks that has been in the area for some time; and consistent with other breeding black ducks studied elsewhere in North Carolina [29]. This aligns historically given the low population density in comparison to northern and central populations within the breeding range, and behaviorally considering the propensity for coastal black ducks to remain local during the non-breeding season [68–70].

Finding several mallard x black duck hybrid females and offspring among our sample set (Fig 3C) was consistent with range-wide molecular analysis finding a generally high hybridization rate of ~25% between black ducks and mallards; though hybrid backcrossing was more likely into mallard than black duck [28]. However, we acknowledge that targeting phenotypically breeding black ducks precluded us from sampling any mallard-looking female, making it necessary for more indiscrete sampling efforts to better understand the extent that wild or game-farm mallards are breeding in the area. In fact, a female mallard was flushed during the study, but her and her brood were not sampled (AJ Hoyt, personal observation). Nevertheless, based on differences in mother-offspring ancestry (Fig 3B), we conclude that breeding black ducks within the study area are mate pairing with other hybrids, wild mallards, and game-farm mallards. In total, we found that non-black duck males were the primary paternal contributor in four of seven nests, with the remaining three pairings resulting in one egg; and thus, likely a result of extra-pair copulation (Table 1). Moreover, in all cases but one, the maternal lineage was inferred as being pure black duck. Though we provide data that makes evident that wild and game-farm mallards and mallard x black duck hybrids are capable of making viable eggs with black ducks, the majority of inferred pairings (i.e., 65%; 13 of 20) were strictly between black duck males and females. What’s more, 80% (16 of 20) of inferred pairings were situations where the female black duck was at least socially monogamous with a male black duck. Together, we conclude that despite the potential for interspecific mating, forms of assortative mating must be strong enough to limit these events [71, 72]. Future work would benefit from mate-choice studies attempting to understand whether females are cueing into specific morphological or other biometric phenotypes when picking their primary mate. Regardless, these interspecific mate-pairings resulted in ~18% (16 of 90) of genotyped eggs with mixed ancestry that undoubtedly will continue to trickle into future generations. We conclude that while the breeding black duck population in North Carolina appears to remain overwhelmingly genetically black duck, any further imbalance in the number of wild or game-farm mallards on the landscape can unfavorably tilt it; and will require continuous genetic monitoring. Moreover, future studies should include the genetic assessment of males breeding in the area as to determine the true overall relatedness among the population’s breeding individuals, and thus, rates of immigration (i.e., gene flow from non-related males). We posit that non-related males are the reason that genetic diversity of the population appears outcrossed (S5 Fig) despite high-levels of relatedness (Fig 2).

Samples were collected primarily from natural islands rather than coastal mainland marshes because only successful nests (i.e., ≥1 egg hatched) were included in the study. We recognize that this regime may introduce some bias regarding the genetic diversity of black ducks in the study area. However, investigation into relatedness among only hatched nests provides the opportunity to explore a connection between population structure and nest success on islands. High interrelatedness amongst sampled broods can in part be explained by natal philopatry that is common among female black ducks [33, 73]. Additionally, past research on other ground nesting waterfowl indicates that nest density in already dense areas may increase over time due to successful females returning to known safe areas and site selection cues from the presence of conspecific nests [74]. If natural islands continue to support high levels of nest success, we can expect to see even higher nest densities on the islands in the future. Further, this would be exacerbated by mammalian predators present in higher densities in mainland marshes depredating nests and thereby pushing females to select islands for nesting [33, 73]. Together, we predict more incidences of nest parasitism, higher interrelatedness among broods and females, and a general dependence on natural islands to support nesting black ducks in the future.

### Deployment of alternative breeding strategies by American black ducks

Despite a rich history of breeding strategy work in waterfowl, our understanding of alternative strategies in upland nesting birds remains limited. Though expected to be seasonally monogamous, female ducks have the potential to deploy three primary alternative breeding strategies that include, extra-pair copulation [14], nest parasitism [15], and brood amalgamation [16]. While simply engaging in nest parasitism does not preclude an individual from being socially and sexually monogamous, taking part or forced extra-pair copulation that leads to fecundity to an alternative mate is. We assessed whether any of the breeding female black ducks engaged in either of the alternative breeding strategies by genotyping their eggs using both mitochondrial and nuclear DNA. First, a combination of unique mtDNA haplotypes and/or nuclear parentage assignment indicative of nest parasitism was only found to have been deployed on two of the nests (nests 10 and 19; S1 Table), whereas 37% (7 of 19) of the sampled nests were multi-paternal as a result of extra-pair copulation; the latter is consistent with earlier work on mallards [26]. Across the seven multi-paternal nests, we found a maximum of two inferred males (Table 1; S1 Table), and in each case the extra-pair copulation resulting in one additional egg. We acknowledge that the true contribution of each paternal lineage cannot be discerned in most cases due to incomplete nuclear sequencing of all the eggs comprising each clutch. Among the nests, nest 10 was most interesting as the inferred father of one of the primary female’s eggs and that of the two parasitizing eggs was the same (Table 1). In this case, the father who was likely socially paired with un-sampled female #3 was able to obtain an extra-pair copulation with AH2 (i.e., primary female of nest 10), while the un-sampled female also parasitized the nest. In general, we provide results consistent with the hypothesis that high nest densities often result in higher rates of extra-pair copulation and/or nest parasitism due to the ease and proximity to alternative mates and other nests, respectively [16, 20, 22–25].

Given the high relatedness and nest proximity among sampled black ducks, we would have posited that nest parasitism would be the dominant alternative breeding strategy deployed by females as a form of kin selection [15, 75]. While we did find evidence for parasitism in two (i.e., ~10.5%) nests, extra-pair copulation was overwhelming the preferred secondary breeding strategies (Table 1). We posit that extra-pair copulation was the major alternative breeding strategy among our sampled black ducks due to a combination of high nest densities providing easier alternative mate access for males [9], and potentially a mixed of strategies to increase fecundity of already successfully breeding females [76]. Ultimately, however, while some proportion of females of many species engage in forms of secondary breeding strategies, the decision to do so appears to be seasonally flexible for each individual [6, 17]. We note that although broods were not tracked further into the season, we predict potentially high rates of brood amalgamation occurring among kin females, which would be an additional alternative post-breeding strategy that could be employed in such populations [16].

## Supporting information

S1 Table Sample information. (XLSX)

S2 Table Relatedness matrix of pair-wise sample Ajk statistics calculated from ddRAD-seq nuclear variation for 19 female and 80 offspring duck samples in North Carolina, USA, 2020–2021. (XLSX)

S1 Fig Sibling relatedness plot of black duck female samples (*N* = 19) from North Carolina, USA, 2020–2021, as estimated in program COLONY. (PDF)

S2 Fig Sibling relatedness plot of black duck offspring samples (*N* = 80) from North Carolina, USA, 2020–2021, as estimated in program COLONY. (PDF)

S3 Fig EDENetworks network of the 20 known and inferred nest groups (Fig 2; Table 1) identified across the 99 North Carolina, USA, 2020–2021, samples, and reconstructed using 269 independent bi-allelic ddRAD-seq autosomal SNPs that met coverage and minimum allele frequency criteria. (PNG)

S4 Fig Offspring sex ratios and total number of eggs per nest collected in coastal North Carolina, 2020–2021. (PNG)

S5 Fig Boxplots of (A) nucleotide diversity (π) and (B) pair-wise estimates of relative differentiation (Φ_ST_) calculated across 80,035 base-pairs of ddRAD-seq autosomal loci among reference American black ducks, wild mallards, and game-farm mallards, and North Carolina, USA samples, 2020–2021.
(PNG)
