# Supplementary figures and images for: Frequency and types of alternative breeding strategies employed by nesting American black ducks in North Carolina
Source: PLoS One. 2023 Feb 21;18(2):e0278905. doi: 10.1371/journal.pone.0278905 (PMC9942969; doi:10.1371/journal.pone.0278905)

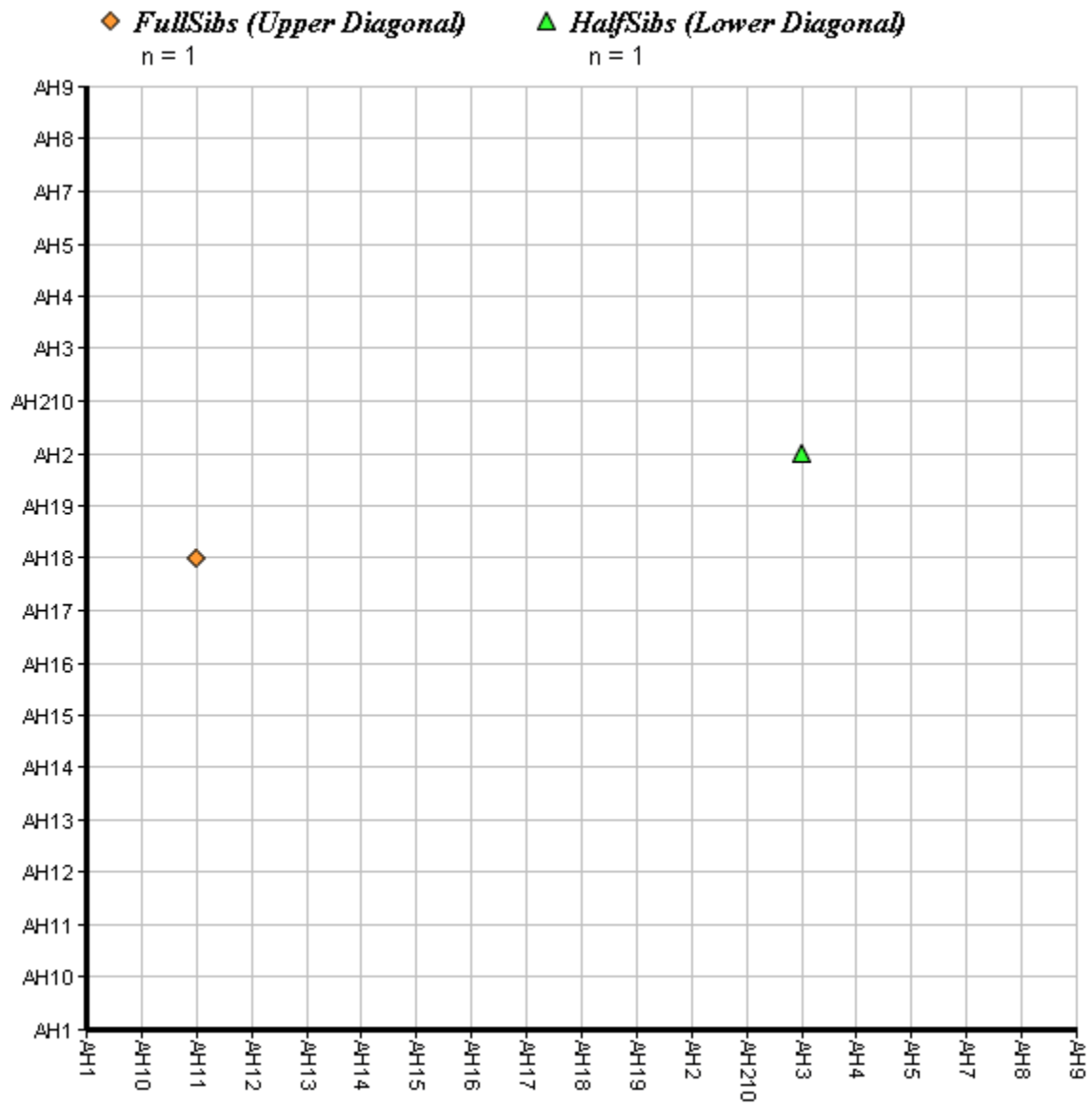

Supplement: S1 Fig — (PDF) [file pone.0278905.s003.pdf]

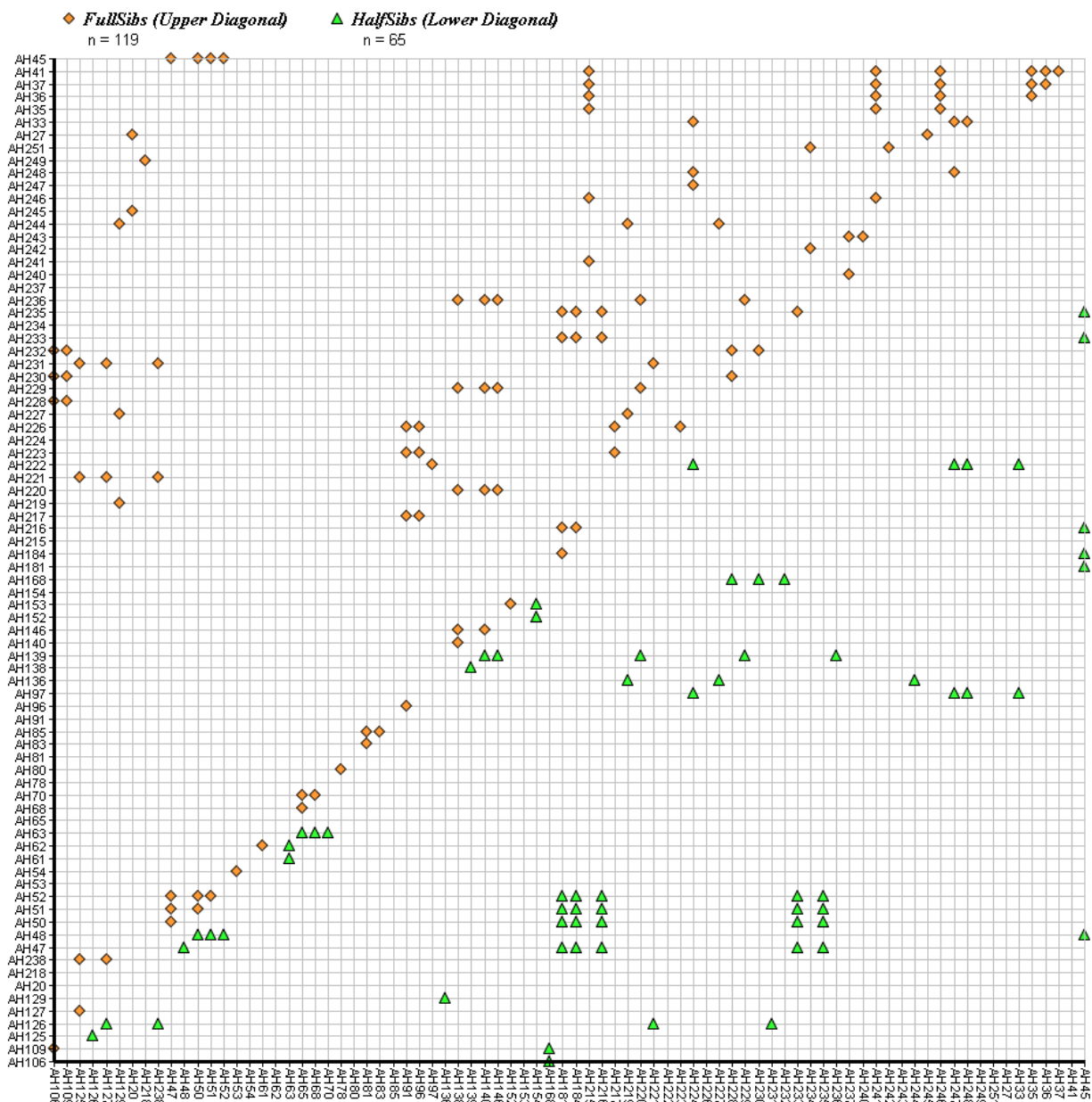

Supplement: S2 Fig — (PDF) [file pone.0278905.s004.pdf]

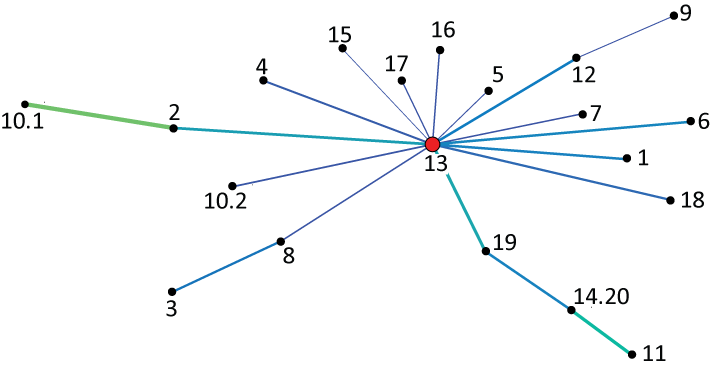

Supplement: S3 Fig — (PNG) [file pone.0278905.s005.png]

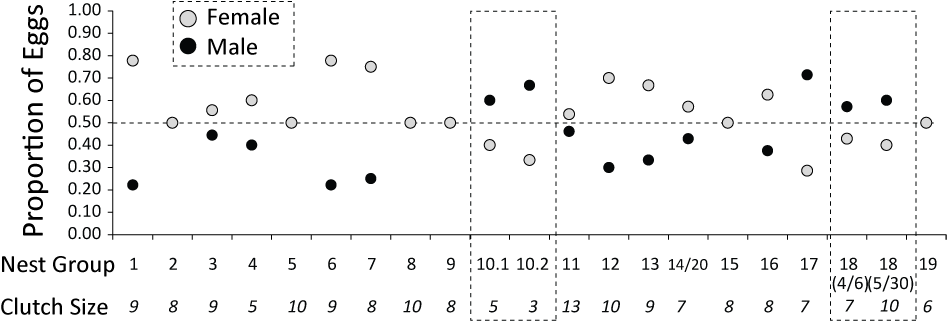

Supplement: S4 Fig — (PNG) [file pone.0278905.s006.png]

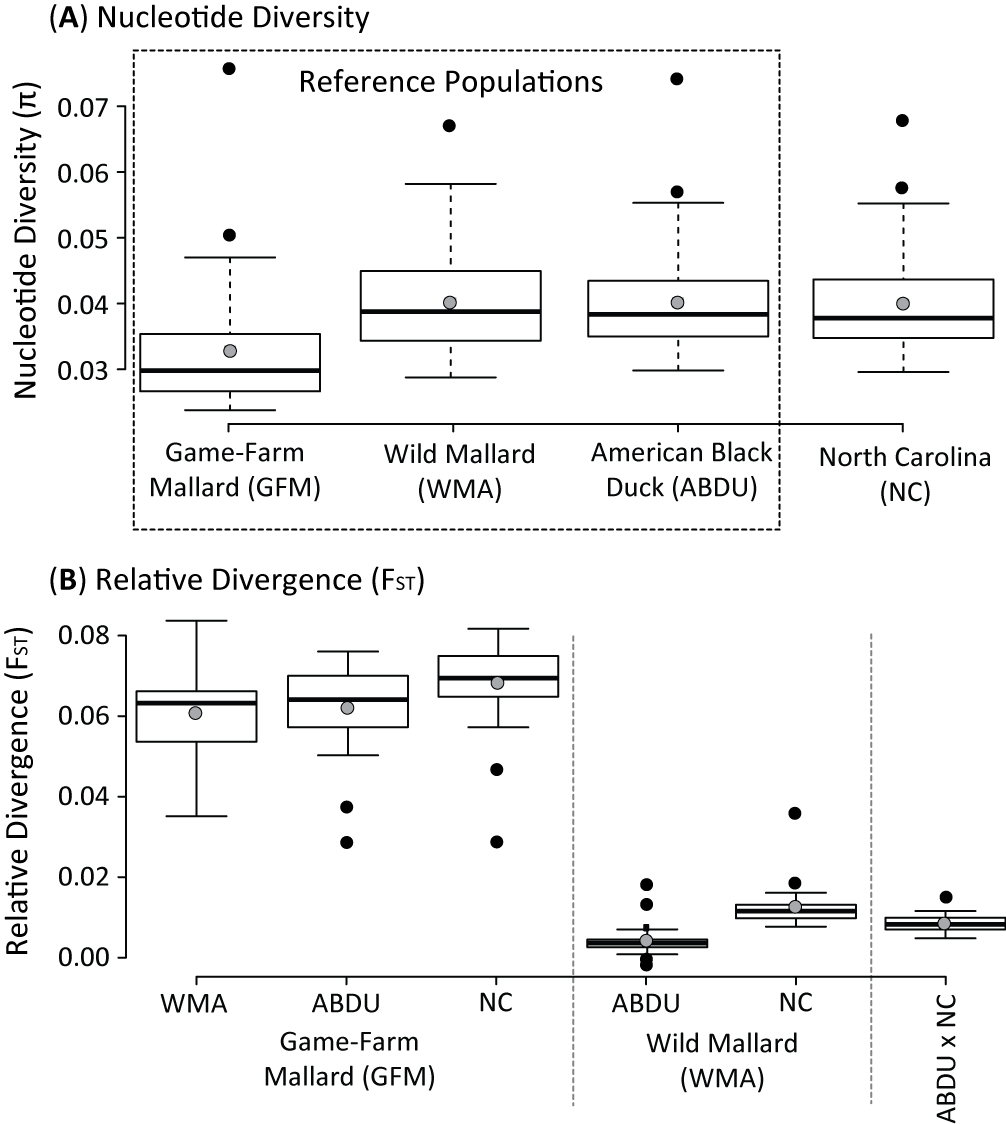

Supplement: S5 Fig — Boxplots of (A) nucleotide diversity (π) and (B) pair-wise estimates of relative differentiation (ΦST) calculated across 80,035 base-pairs of ddRAD-seq autosomal loci among reference American black ducks, wild mallards, and game-farm mallards, and North Carolina, USA samples, 2020–2021. (PNG) [file pone.0278905.s007.png]
